# SARS-CoV-2 infection is detrimental to pregnancy outcomes after embryo transfer in IVF/ICSI: a prospective cohort study

**DOI:** 10.1186/s12916-024-03336-9

**Published:** 2024-03-18

**Authors:** Yuan Li, Qi Zhao, Shujuan Ma, Sha Tang, Guangxiu Lu, Ge Lin, Fei Gong

**Affiliations:** 1 Clinical Research Center for Reproduction and Genetics in Hunan Province, Reproductive and Genetic Hospital of CITIC-XIANGYA, No. 567 Tongzipo West Road, Yuelu District, Hunan Changsha, 410008 China; 2 Laboratory of Reproductive and Stem Cell Engineering, Key Laboratory of National Health and Family Planning Commission, Central South University, Hunan Changsha, 410008 China

**Keywords:** SARS-CoV-2 infection, Embryo transfer, IVF/ICSI, Pregnancy outcomes

## Abstract

**Background:**

To explore whether SARS-CoV-2 infection affects the pregnancy outcomes of assisted reproductive techniques (ART).

**Methods:**

A prospective cohort study recruited patients for embryo transfer from December 01, 2022, to December 31, 2022. All patients were closely followed up for SARS-CoV-2 infection after embryo transfer. The SARS-CoV-2 “diagnosed group” was defined as RNA or antigen-positive. The SARS-CoV-2 “suspected infection group” was defined as having apparent SARS-CoV-2 symptoms without an RNA or antigen test, while the “uninfected group” was defined as having a negative SARS-CoV-2 RNA or antigen test and no SARS-CoV-2 symptoms.

**Results:**

A total of 1330 patients participated in the study, 687 of whom were in the SARS-CoV-2 diagnosed group, 219 in the suspected infection group, and 424 in the uninfected group. There was no significant difference in basic characteristics among the three groups. The clinical pregnancy rate was 68% in the SARS-CoV-2 diagnosed group, 63% in the uninfected group, and 51% in the suspected infection group (*P* < 0.001). The ongoing pregnancy rate was 58% in the SARS-CoV-2 diagnosed group, 53% in the uninfected group, and 45% in the suspected infection group (*P* < 0.001). Upon analyzing the factors influencing clinical pregnancy, it was found that suspected infection (odds ratio [OR] 0.618, 95% CI 0.444–0.862, *P* = 0.005) and the short time (≤ 22 days) between embryo transfer and SARS-CoV-2 infection (OR 3.76, 95% CI 1.92–8.24, *P* < 0.001) were not conducive to clinical pregnancy. In addition, the concurrent presence of fever and dizziness/headache SARS-CoV-2 symptoms (OR 0.715, 95% CI 0.526–0.972, *P* = 0.032) decreased the clinical pregnancy rate. However, vaccination administered 2–3 times (OR 1.804, 95% CI 1.332–2.444, *P* < 0.001) was associated with an improvement in clinical pregnancy rate.

**Conclusions:**

This prospective cohort study shows that SARS-CoV-2 infection in a short period of time after embryo transfer is not conducive to clinical pregnancy. Reproductive physicians should advise patients to avoid SARS-CoV-2 infection shortly after embryo transfer. Meanwhile, women should be encouraged to vaccinate at least 2–3 times before embryo transfer or pregnancy.

**Supplementary Information:**

The online version contains supplementary material available at 10.1186/s12916-024-03336-9.

## Background

SARS-CoV-2 infection affects not only the health of people all over the world but also the reproductive health of women. The viral spike (S) protein of SARS-CoV-2 plays a crucial role in virus infection. The described mechanism involves viral spike (S) protein-mediated recognition and binding to the angiotensin 1-converting enzyme 2 (ACE2) receptor on the host cell, after which the protease transmembrane serine protease 2 (TMPRSS2) cleaves the S protein to facilitate cell entry [1, 2]. An alternative ACE2-independent mechanism exists, with basigin (BSG or CD147) acting as the cellular receptor and cysteine protease cathepsin L (CTSL) as the protease, potentially mediating SARS-CoV-2 cell entry [1, 2]. Tissues and cells that express these receptors and proteases, particularly those with high co-expression of ACE2 and TMPRSS2, are likely to be more vulnerable to SARS-CoV-2 infection. Previous studies detected low percentages of SARS-CoV-2 virus in female vaginal secretions (5.7–12.5%) and cervical secretions (10.53%) [3–5]. Due to the limitation of virus detection technology, the current clinical practice often indirectly infers the impact of SARS-CoV-2 infection on different organs by detecting SARS-CoV-2 receptors in different tissues [6]. Previous literature has confirmed that SARS-CoV-2 receptors (classical/nonclassical) are expressed in the female reproductive system (endometrium, ovaries) [1, 7, 8], meaning there is a theoretical possibility that the female reproductive system may be affected by viral infections.

Previous research has established that SARS-CoV-2 infection during controlled ovarian stimulation (COS) may influence embryo development [9, 10]. Some studies have also confirmed that SARS-CoV-2 infection in pregnancy is associated with increases in severe maternal morbidity, mortality, and neonatal complications [11, 12]. It remains controversial whether SARS-CoV-2 infection has adverse effects on early pregnancy outcomes [13, 14]. Previously published articles have shown that the recovery from SARS-CoV-2 infection does not affect pregnancy outcomes in fresh cycles with embryo transfer [15]. Additionally, SARS-CoV-2 vaccination has been found to not negatively affect ovarian function during assisted reproductive technology (ART) outcomes [16, 17]. Despite the available literature, there is a lack of research on whether SARS-CoV-2 infection affects early pregnancy outcomes shortly after an embryo transfer in in vitro fertilization (IVF) or intracytoplasmic sperm injection (ICSI). Therefore, the main purpose of this study is to explore whether SARS-CoV-2 infection in the short term after embryo transfer is detrimental to pregnancy outcomes.

## Methods

### Study population and design

SARS-CoV-2 control measures were fully lifted in China in December 2023. Consequently, we aimed to study the impact of SARS-CoV-2 infection on the clinical pregnancy rate of women receiving ART during the lifting of control measures for the SARS-CoV-2 epidemic situation. The study was conducted as a single-center research project in a tertiary hospital. This study is a prospective cohort study, in which recruited patients underwent embryo transfer in the IVF/ICSI/frozen-thawed embryo (FET) cycle from December 1, 2022, to December 31, 2022. The inclusion criteria were as follows: (1) patients undergoing IVF/ICSI/FET cycle and embryo transfer from December 1, 2022, to December 31, 2022; (2) patients willing to provide information on SARS-CoV-2 infection and vaccination; and (3) patients willing to undergo SARS-CoV-2 RNA or antigen tests. The exclusion criterion was patients with a previous history of SARS-CoV-2 infection before embryo transfer.

The diagnosed group of SARS-CoV-2 infection refers to individuals who tested positive for RNA by using reverse transcriptase polymerase chain reaction (RT-PCR) assays on oropharyngeal swab or tested positive for antigen on nasopharynx swab. The uninfected group refers to patients who tested negative for SARS-CoV-2 RNA or antigen testing and did not show any symptoms of SARS-CoV-2. All patients from the uninfected group underwent SARS-CoV-2 RNA or antigen testing, and the results were negative for all of them. Some patients were unable to undergo SARS-CoV-2 RNA or antigen testing when the epidemic broke out in China, despite displaying obvious symptoms of SARS-CoV-2 infection and having family members test positive for SARS-CoV-2. To ensure a more objective analysis of the impact of SARS-CoV-2 infection on pregnancy outcomes, these patients were classified and analyzed along with the SARS-CoV-2 suspected infection group. There has been corresponding literature on this classification in the past [14].

### Embryo transfer

#### Fresh embryo transfer (fresh ET) cycle

The ovarian-stimulation protocols were carried out according to the hospitals’ protocol [18]. Ovulation was triggered by administering 5000–10,000 IU of human chorionic gonadotropin (hCG) when two-thirds of the follicles reached 18 mm in size. Transvaginal ultrasound-guided oocyte retrieval was performed 34–36 h post-hCG administration. Oocytes were fertilized by IVF or ICSI 4–6 h after oocyte retrieval. Normal fertilization was confirmed by the presence of two pronuclei and two polar bodies at 16–18 h after insemination or injection. The resulting embryos were cultured in G1.5/G2.5 sequential media (Vitrolife) until reaching the blastocyst stage. The culturing process took place in a COOK mini-incubator at 37 °C, with a humidified atmosphere of 6% CO_2_, 5% O_2_, and 89% N_2_. Two cleavage-stage embryos, one good-quality blastocyst, or one to two non-good blastocysts (depending on the patient’s condition or preferences) were selected and transferred into the uterus.

#### Frozen thawed embryo transfer (FET) cycle

All embryos were graded before freezing in a FET cycle. On day 3, embryos were scored using Puissant’s criterion, while blastocysts were graded according to Gardner and Schoolcraft’s system on day 5, 6, or 7. All embryos were vitrified and thawed using a Kitazato vitrification kit (Kitazato Biopharma, Shizuoka, Japan) in combination with closed high-security vitrification Straws (Cryo Bio System, France). Embryos were thawed on the day of transfer. Thawed embryos were prioritized based on best quality before freezing. The thawed embryos were then transferred to G2.5 medium and cultured for 2–6 h. Embryos were considered suitable for transfer when more than half of the blastomeres were recovered or the blastocyst re-expanded. Depending on the patient’s condition or preference, the transfer involved the placement of two cleavage-stage embryos, one good-quality blastocyst, or one to two non-good blastocysts.

### Outcome assessments

The primary outcome was clinical pregnancy, defined as the presence of at least one gestational sac in the uterine cavity, confirmed with ultrasound, approximately 28 days after embryo transfer. The secondary outcomes included early miscarriage (loss of clinical pregnancy before the 12th gestational week) and ongoing pregnancy (presence of at least one gestational sac in the uterine cavity on ultrasound at the 12th gestational week). Biochemical pregnancy referred to a positive serum β-hCG level with no gestational sac detected via ultrasound. Ectopic pregnancy referred to a gestational sac that appeared outside the uterine cavity. All clinical outcomes were defined according to the International Glossary on Infertility and Fertility Care [19].

### Statistical analysis

All analyses were performed using the R software (R version 4.2.2). Continuous variables were reported as mean ± standard deviation. Analysis of variance was used for normally distributed values, and the Kruskal–Wallis rank sum test was used for skewed data. Categorical variables were summarized using frequencies and percentages and were analyzed using Pearson’s chi-square or Fisher’s exact test, as appropriate. All pregnancy outcomes were compared among the three groups using Fisher’s exact test. However, only the primary pregnancy outcome (clinical pregnancy) was analyzed using logistic regression. The odds ratios (OR) of clinical pregnancy in comparison with non-pregnancy were evaluated by logistic regression analysis. Significance tests were two-tailed and conducted at the 0.05 significance level.

## Results

A total of 1393 patients underwent embryo transfer between December 1 and December 31, 2022. Out of these, 63 patients had a history of SARS-CoV-2 infection before embryo transfer (time interval 1–109 days) and were excluded from the analysis. The final dataset included 1330 patients, divided into the following three groups: 51.6% (*n* = 687) were in the SARS-CoV-2 diagnosed group, 16.4% (*n* = 219) in the SARS-CoV-2 suspected infection group, and 32% (*n* = 424) in the uninfected group. The basic characteristics of the patients in the three groups, including age and the presence of special diseases such as polycystic ovary syndrome (PCOS), endometriosis, adenomyosis, intrauterine adhesions, obesity, and diabetes, as well as the rate of top-quality embryo transfer, were not significantly different. The clinical pregnancy rate was 68% in the SARS-CoV-2 diagnosed group, 63% in the uninfected group, and 51% in the suspected infection group (*P* < 0.001) (Table 1). The ongoing pregnancy rate was 58% in the SARS-CoV-2 diagnosed group, 53% in the uninfected group, and 45% in the suspected infection group (*P* < 0.001) (Table 1). There was no significant difference in biochemical pregnancy rate, early miscarriage rate, or ectopic pregnancy rate (Table 1).

**Table 1:** Baseline characteristics and pregnancy outcomes of the SARS-CoV-2 diagnosed group, suspected infection group and uninfected group

	SARS-CoV-2 uninfected group (*n* = 424)	SARS-CoV-2 diagnosed group (*n* = 687)	SARS-CoV-2 suspected infection group (*n* = 219)	*P* value
Age	32.9 (4.7)	32.6 (4.4)	32.9 (4.7)	0.6
BMI	22.1 (2.6)	21.9 (2.7)	22.2 (2.6)	0.10
Infertility type				0.5
Primary	25% (106/418)	28% (190/682)	24% (52/213)	
Secondary	75% (312/418)	72% (492/682)	76% (161/213)	
Special diseases				
PCOS	21% (89/424)	19% (132/687)	21% (45/219)	0.8
Endometriosis	7% (28/424)	7% (50/687)	4% (8/219)	0.2
Adenomyosis	22% (93/424)	23% (160/687)	19% (41/219)	0.4
Moderate to severe intrauterine adhesions	21% (91/424)	17% (118/687)	19% (42/219)	0.2
Untreated hydrosalpinx	7% (28/424)	6% (44/687)	7% (16/219)	0.9
Obesity	2% (10/424)	4% (25/687)	1% (2/219)	0.083
Diabetes	0% (2/424)	1% (5/687)	1% (2/219)	0.7
More than two mixed diseases	19% (80/424)	16% (113/687)	16% (34/219)	0.5
Top-quality embryo transfer rate	63% (267/424)	65% (446/687)	65% (142/219)	0.8
Embryo transfer cycle				0.3
Fresh cycle	23% (96/424)	25% (175/687)	21% (46/219)	
FET cycle	77% (328/424)	75% (512/687)	79% (173/219)	
Endometrial thickness on the day of embryo transfer	11 (2)	12 (2)	12 (2)	0.073
Pregnancy outcomes				
Clinical pregnancy rate	63% (267/424)	68% (464/687)	51% (111/219)	< 0.001
Biochemical pregnancy rate	10% (41/424)	10% (70/687)	12% (26/219)	0.7
Ectopic pregnancy rate	1% (6/424)	1% (4/687)	0% (1/219)	0.3
Early miscarriage rate	13% (34/263)	13% (58/459)	10% (11/109)	0.7
Ongoing pregnancy rate	53% (220/417)	58% (394/678)	45% (96/215)	0.002

Analysis of vaccination and SARS-CoV-2 infection information showed that the SARS-CoV-2 diagnosed group and suspected infection group had a comparable vaccination rate (83% vs 84%). The proportion of patients with a shorter time interval (14–21 days) between embryo transfer and SARS-CoV-2 infection was higher in the SARS-CoV-2 suspected infection group (38% vs 25%, *P* < 0.001) than in the diagnosed group (Table 2). There were higher proportions of symptoms with fever (60% vs 68%, *P* = 0.032), dizziness/headache (34% vs 43%, *P* = 0.022), or loss of appetite (19% vs 28%, *P* = 0.007) in the suspected infection group compared to the diagnosed group (Table 2). Although not statistically significant, the incidence of cough (76% vs 74%), sore throat (39% vs 34%), and muscle soreness (45% vs 47%) in the SARS-CoV-2 suspected infection group was higher than in the diagnosed group (Additional file 1: Table S1).

**Table 2:** Vaccination and SARS-CoV-2 infection information during SARS-CoV-2: diagnosed group, suspected infection group and uninfected group

	SARS-CoV-2 uninfected group (*n* = 424)	SARS-CoV-2 diagnosed group (*n* = 687)	SARS-CoV-2 suspected infection group (*n* = 219)	*P* value
Vaccination rate	77% (289/374)	83% (568/684)	84% (184/218)	0.034
Vaccination frequency				< 0.001
1	1% (6/409)	2% (14/668)	1% (3/211)	
2	25% (103/409)	32% (213/668)	36% (76/211)	
3	44% (178/409)	51% (341/668)	49% (104/211)	
4	0% (1/409)	0% (1/668)	0% (1/211)	
Time from the last vaccination to infection	NA (NA)	382.7 (127.2)	391.0 (135.5)	0.4
Time interval between embryo transfer and infection				0.001
1 (< 7 days)		22% (153/687)	21% (46/219)	
2 (7–13 days)		39% (271/687)	31% (67/219)	
3 (14–20 days)		25% (171/687)	38% (84/219)	
4 (≥ 21 days)		13% (92/687)	10% (22/219)	
SARS-CoV-2 infection symptoms				
Fever		60% (412/687)	68% (149/219)	0.032
Dizziness/headache		34% (236/687)	43% (94/219)	0.022
Loss of appetite		19% (132/687)	28% (61/219)	0.007

Logistic regression analysis was performed to explore the factors affecting clinical pregnancy. There were no statistically significant differences in the basic characteristics between the clinical pregnancy group and the non-pregnancy group (Additional file 2: Table S2). However, vaccination (OR 1.513, 95% CI 1.134–2.019, *P* = 0.005), especially three doses of the vaccine (OR 1.804, 95% 1.332–2.444, *P* < 0.001), can help improve the clinical pregnancy rate compared to unvaccinated individuals (Table 3). Suspected infection status decreased the clinical pregnancy rate by 38.2% (OR 0.618, 95% CI 0.444–0.862, *P* = 0.005) compared to the uninfected group. A longer time interval between embryo transfer and SARS-CoV-2 infection was associated with a higher clinical pregnancy rate (OR 1.022, 95% CI 1.001–1.043, *P* = 0.042) (Table 3). Receiver operating characteristic (ROC) analysis identified the cutoff value of 22 days as a predictor for the interval time between embryo transfer and SARS-CoV-2 infection (OR 3.76 95% CI 1.92–8.24, *P* < 0.001) (Additional file 3: Fig. S1, Additional file 4: Table S3). Although there was no statistical difference in the overall symptoms of SARS-CoV-2 infection, the overall incidence of SARS-CoV-2 symptoms in the pregnancy group was lower (Additional file 5: Table S4). However, SARS-CoV-2 symptoms with the occurrence of fever and dizziness/headache decreased the clinical pregnancy rate by 28.5% (OR 0.715, 95% CI 0.526–0.972, *P* = 0.032) (Table 3).

**Table 3:** Vaccination and SARS-CoV-2 infection information between the pregnancy group and the non-pregnancy group

	Non-pregnancy group (*N* = 486)	Pregnancy group (*N* = 826)	*P* value	OR	*P* value
Vaccination rate	77% (357/461)	84% (670/799)	0.005	1.513 (1.134–2.019)	0.005
Vaccination frequency			0.002		
0	24% (113/471)	16% (130/799)		Ref	
1	2% (11/471)	2% (12/799)		0.948 (0.403–2.232)	0.903
2	31% (147/471)	30% (242/799)		1.431 (1.034–1.98)	0.031
3	42% (199/471)	52% (413/799)		1.804 (1.332–2.444)	< 0.001
4	0% (1/471)	0% (2/799)		1.738 (0.156–19.427)	0.653
Time from last vaccination to infection (days)	385.2 (134.9)	385.6 (126.6)	0.8	1 (0.999–1.001)	0.97
SARS-CoV-2 infection state			< 0.001		
0 (uninfected group)	32% (157/486)	32% (261/826)		Ref	
1 (diagnosed group)	46% (222/486)	55% (455/826)		1.233 (0.956–1.591)	0.107
2 (suspected infection group)	22% (107/486)	13% (110/826)		0.618 (0.444–0.862)	0.005
Time interval between embryo transfer and infection	11.4 (6.2)	12.4 (6.8)	0.068	1.022 (1.001–1.043)	0.042
SARS-CoV-2 infection symptoms					
Fever and dizziness/headache	29% (97/329)	23% (130/565)	0.032	0.715 (0.526–0.972)	0.032
Fever and loss of appetite	17% (55/329)	17% (94/565)	> 0.9	0.994 (0.691–1.432)	0.975
Dizziness/headache and loss of appetite	13% (44/329)	12% (68/565)	0.6	0.886 (0.59–1.33)	0.56

## Discussion

This is the first large sample prospective cohort study to explore the impact of SARS-CoV-2 infection in the short term after embryo transfer on pregnancy outcomes. The findings suggest that symptomatic SARS-CoV-2 infection shortly after embryo transfer is not conducive to clinical pregnancy.

Existing published articles have primarily focused on whether SARS-CoV-2 infection during pregnancy affects obstetric outcomes or early miscarriage rate [11–14, 20]. Currently, the adverse effects of obstetric outcomes are relatively clear, with women diagnosed with COVID-19 being at an increased risk of a composite maternal morbidity and mortality index [11, 12]. However, there is ongoing controversy regarding the early miscarriage rate [13, 14, 20]. Moreover, in the prospective cohort, 18 (1.8%) women had SARS-CoV-2 antibodies in the serum from the double test, which is suggestive of SARS-CoV-2 infection, in early pregnancy [13]. This retrospective case–control study suggested that the early miscarriage rate was nearly 50%, although the population selection may have affected the results [20]. The majority of these studies have focused on non-assisted reproductive technology (non-ART) pregnancies, making it challenging to accurately determine the interval time between SARS-CoV-2 infection and embryo implantation or to determine the impact on women receiving ART [14].

The mechanism of adverse effects of SARS-CoV-2 infection on pregnancy outcomes in the short term after embryo transfer is not fully understood. However, previously published articles suggest that two aspects may be considered. The first mechanism is that SARS-CoV-2 directly infects the embryo, affecting embryonic development. Studies analyzing RNA sequencing data of donated zygotes and blastocysts show that early embryos and late blastocysts express ACE2, while TMPRSS2 is expressed only in late blastocysts, indicating the co-expressed two types of receptors in late blastocysts [21]. Another study utilizing donated human gametes evaluated the expression of SARS-CoV-2 receptor protein on embryos and found that the 5th- and 7th-day blastocysts showed the expression of ACE2 and BSG on the trophoblast and inner cell mass cell membrane, suggesting that SARS-CoV-2 could potentially infect embryos [22]. When human blastocysts are exposed to SARS-CoV-2, both trophoblast cells and inner cell mass cells are infected, displaying signs of cellular degeneration, indicating the pathogenic effect of infection [23]. The zonal pellucida seems to have a protective effect against SARS-CoV-2 infection [23]. During embryo implantation, as the blastocyst hatches and the protective effect of the zona pellucida is removed, the embryo implantation process may be affected by the pathogenic effect of the SARS-CoV-2 virus. The second mechanism involves the potential impact of SARS-CoV-2 infection on maternal immune status. Previous literature suggests that immune cells in the peripheral blood after SARS-CoV-2 infection undergo two-way changes: activation of NK cells and excessive depletion of NK and T cells [24]. The immune status experiences the process of innate immunity, adaptive immunity, and immune tolerance after SARS-CoV-2 infection [24–27]. Previous studies have found that SARS-CoV-2 infection leads to severe peripheral lymphocyte reduction with decreased numbers of CD4 + T cells, CD8 + T cells, NK cells, and B cells [28]. A study on patients with long COVID found altered T cells, including depleted T cells and reduced CD4 + and CD8 + effector memory cells persisting for 13 months [29]. A comprehensive study comparing patients with long COVID to uninfected individuals and infected individuals without long COVID found increased numbers of non-classical monocytes, activated B cells, and IL-4- and IL-6-secreting CD4 + T cells, as well as exhausted T cells in individuals with long COVID at a median of 14 months after infection [29, 30]. Therefore, SARS-CoV-2 infection may affect clinical pregnancy outcomes by interfering with maternal immune balance.

Our research results suggest that the SARS-CoV-2 diagnosed group does not affect clinical pregnancy significantly, while the suspected infection group was not conducive to pregnancy outcomes. The main difference between these two groups was the presence of symptoms. Analysis of the SARS-CoV-2 diagnosis group found that 95% of the patients had SARS-CoV-2 symptoms, making it impossible to further analyze SARS-CoV-2 infection based on asymptomatic cases. While only fever and dizziness/headache symptoms were found to be not conducive to clinical pregnancy, the overall incidence of symptoms in the non-pregnant group was still significantly higher than that in the pregnant group (Table 3). Based on these observations, we speculated that the symptoms of SARS-CoV-2 infection may indicate the maternal immune response to SARS-CoV-2 infection. Meanwhile, implantation failure is often accompanied by a shift in the phenotypes of immune cells, with a bias toward effector T cells, overactivated or under-activated uNK cells, M1 (pro-inflammatory) macrophages, or immunogenic dendritic cells [31]. Previous literature suggests that severe SARS-CoV-2 infection is accompanied by a T cell response with a delayed cytotoxic reaction [27]. Therefore, we speculated that SARS-CoV-2 infection may lead to changes in immune status at the early stage of embryo implantation, leading to unfavorable conditions for successful embryo implantation. Thus, the possible mechanism of SARS-CoV-2 infection in the short term (≤ 22 days) after embryo transfer is more concerned with changes in the immune state. Moreover, the vaccination for two to three times improves clinical pregnancy, which further supports the above supposition.

The main advantage of this study is the relatively large sample prospective cohort study. In addition, this research explores the real data of the first round of the SARS-CoV-2 infection outbreak in China. It is helpful to understand the influence of SARS-CoV-2 infection on pregnancy outcomes after embryo transfer, and our data suggest that there is no difference in baseline characteristics related to pregnancy outcomes, which makes the conclusion more reliable.

This study explores the impact of SARS-CoV-2 infection symptoms on pregnancy outcomes, which is another advantage as it is a more comprehensive evaluation of the possible impact of SARS-CoV-2 infection on women of childbearing age. Yet, it is also a limitation because all SARS-CoV-2 symptoms are recalled based on oral descriptions provided by patients during consultations. Not all SARS-CoV-2 infection symptoms can be accurately defined through medical testing indicators such as the specific degree of dizziness/headache, which makes exploring the value of SARS-CoV-2 infection symptoms on pregnancy outcomes difficult.

## Conclusions

This study confirms that symptomatic SARS-CoV-2 infection shortly after embryo transfer is not conducive to clinical pregnancy. Given this, reproductive physicians should give infertile women appropriate medical advice by recommending them to avoid SARS-CoV-2 infection as much as possible after embryo transfer and that two to three or more doses of SARS-CoV-2 vaccine before pregnancy may help reduce the impact of SARS-CoV-2 infection.

### Supplementary Information

**Additional file 1:** **Table S1.** SARS-CoV-2 infection symptoms between the SARS-CoV-2 diagnosed group and suspected infection group.

**Additional file 2:** **Table S2.** Baseline characteristics between the pregnancy group and non-pregnancy group.

**Additional file 3:** **Fig. S1.** ROC curve analysis of time interval between embryo transfer and infection in predicting clinical pregnancy. The area under the ROC curve was 0.537 (95% CI, 0.498–0.575), while the optimal cutoff value was 22 days (with a sensitivity of 97.3% and specificity of 9.6%).

**Additional file 4:** **Table S3.** Proportions of the time interval between embryo transfer and infection in the pregnancy group and non-pregnancy group.

**Additional file 5:** **Table S4.** SARS-CoV-2 infection symptoms between the pregnancy group and non-pregnancy group.

## Data Availability

The datasets used and analysed during the current study are available from the corresponding author on reasonable request.

## Abbreviations

ACE2: Angiotensin 1-converting enzyme 2
ART: Assisted reproductive techniques
BSG: Basigin
COS: Controlled ovarian stimulation
CTSL: Cysteine protease cathepsin L
FET: Frozen-thawed embryo transfer
fresh ET: Fresh embryo transfer
hCG: Human chorionic gonadotropin
ICSI: Intracytoplasmic sperm injection
IVF: In vitro fertilization
OR: Odds ratios
PCOS: Polycystic ovary syndrome
RT-PCR: Reverse transcriptase polymerase chain reaction
TMPRSS2: Protease transmembrane serine protease 2
