# Supplementary material for: SARS-CoV-2 infection is detrimental to pregnancy outcomes after embryo transfer in IVF/ICSI: a prospective cohort study
Source: BMC Med. 2024 Mar 18;22:124. doi: 10.1186/s12916-024-03336-9 (PMC10949839; doi:10.1186/s12916-024-03336-9)
Supplement: Supplementary file 1 — Additional file 1: Table S1. SARS-CoV-2 infection symptoms between the SARS-CoV-2 diagnosed group and suspected infection group. [file 12916_2024_3336_MOESM1_ESM.docx]

Table S1: SARS-CoV-2 infection symptoms between the **SARS-CoV-2 diagnosed group and** suspected infection group

|  | SARS-CoV-2 **diagnosed group**  (n=687) | SARS-CoV-2 suspected infection group  (n=219) | P value |
| --- | --- | --- | --- |
| Cough/dry cough | 74% (510/687) | 76% (167/219) | 0.5 |
| Sore throat | 39% (265/687) | 34% (74/219) | 0.2 |
| Chest tightness/chest pain | 3% (23/687) | 5% (11/219) | 0.3 |
| Muscle soreness/bloating | 45% (308/687) | 47% (103/219) | 0.6 |
| Bone/Arthralgia | 9% (61/687) | 9% (20/219) | ＞0.9 |
| Diarrhea and abdominal pain | 4% (28/687) | 7% (15/219) | 0.093 |
| Nausea and vomiting | 8% (52/687) | 6% (14/219) | 0.6 |
| Fatigue | 18% (124/687) | 21% (47/219) | 0.3 |
| Bitter taste/decreased taste perception | 17% (117/687) | 21% (45/219) | 0.2 |
| Hypoosmia | 8% (57/687) | 8% (17/219) | 0.8 |
| Eye pain/easy tearing/Conjunctivitis | 3% (24/687) | 2% (5/219) | 0.5 |
| Skin symptoms such as Hives and herpes | 1% (7/687) | 2% (4/219) | 0.3 |
| Dyspnea | 1% (5/687) | 0% (1/219) | ＞0.9 |
| Severe symptoms such as hypoxemia and acute respiratory distress syndrome | 0% (1/687) | 0% (0/219) | ＞0.9 |
