# Supplementary material for: SARS-CoV-2 infection is detrimental to pregnancy outcomes after embryo transfer in IVF/ICSI: a prospective cohort study
Source: BMC Med. 2024 Mar 18;22:124. doi: 10.1186/s12916-024-03336-9 (PMC10949839; doi:10.1186/s12916-024-03336-9)
Supplement: Supplementary file 2 — Additional file 2: Table S2. Baseline characteristics between the pregnancy group and non-pregnancy group. [file 12916_2024_3336_MOESM2_ESM.docx]

Table S2.**Baseline Characteristics between the pregnancy group and non-pregnancy group**

|  | Non-pregnancy group(N = 486) | Pregnancy group(N = 826) | P value | OR | P value |
| --- | --- | --- | --- | --- | --- |
| Age | 32.5 (4.4) | 32.8 (4.7) | 0.2 | 1.015 ( 0.99 - 1.04 ) | 0.243 |
| BMI | 22.1 (2.7) | 21.9 (2.7) | 0.2 | 0.978 ( 0.938 - 1.02 ) | 0.298 |
| Infertility type |  |  | 0.4 | 0.901 ( 0.697 - 1.164 ) | 0.424 |
| Primary | 25% (122/481) | 27% (223/814) |  |  |  |
| Secondary | 75% (359/481) | 73% (591/814) |  |  |  |
| Special diseases |  |  |  |  |  |
| PCOS | 23% (112/486) | 19% (153/826) | 0.049 | 0.759 ( 0.577 - 0.999 ) | 0.049 |
| Endometriosis | 7% (32/486) | 7% (54/826) | ＞0.9 | 0.992 ( 0.631 - 1.56 ) | 0.974 |
| Adenomyosis | 23% (111/486) | 21% (177/826) | 0.6 | 0.921 ( 0.704 - 1.206 ) | 0.551 |
| Moderate to severe intrauterine adhesions | 18% (87/486) | 19% (160/826) | 0.5 | 1.102 ( 0.825 - 1.471 ) | 0.511 |
| Untreated hydrosalpinx | 7% (35/486) | 6% (52/826) | 0.7 | 0.866 ( 0.555 - 1.35 ) | 0.524 |
| Obesity | 3% (13/486) | 3% (23/826) | ＞0.9 | 1.042 ( 0.523 - 2.077 ) | 0.907 |
| Diabetes | 0% (2/486) | 1% (7/826) | 0.5 | 2.068 ( 0.428 - 9.997 ) | 0.366 |
| More than two mixed diseases | 19% (90/486) | 16% (135/826) | 0.3 | 0.86 ( 0.641 - 1.153 ) | 0.313 |
| Top-quality embryo transfered rate | 63% (304/486) | 66% (542/826) | 0.3 | 1.143 ( 0.905 - 1.443 ) | 0.263 |
| Embryo transfer cycle |  |  | 0.5 | 0.919 ( 0.706 - 1.197 ) | 0.531 |
| Fresh cycle | 23% (112/486) | 25% (203/826) |  |  |  |
| FET cycle | 77% (374/486) | 75% (623/826) |  |  |  |
| Endometrial thickness on the day of embryo transfer | 12 (2) | 12 (2) | 0.5 | 0.988 ( 0.93 - 1.05 ) | 0.699 |
