# Supplementary material for: SARS-CoV-2 infection is detrimental to pregnancy outcomes after embryo transfer in IVF/ICSI: a prospective cohort study
Source: BMC Med. 2024 Mar 18;22:124. doi: 10.1186/s12916-024-03336-9 (PMC10949839; doi:10.1186/s12916-024-03336-9)
Supplement: Supplementary file 3 — Additional file 3: Fig. S1. ROC curve analysis of time interval between embryo transfer and infection in predicting clinical pregnancy. The area under the ROC curve was 0.537 (95% CI, 0.498–0.575), while the optimal cutoff value was 22 days (with a sensitivity of 97.3% and specificity of 9.6%). [file 12916_2024_3336_MOESM3_ESM.docx]

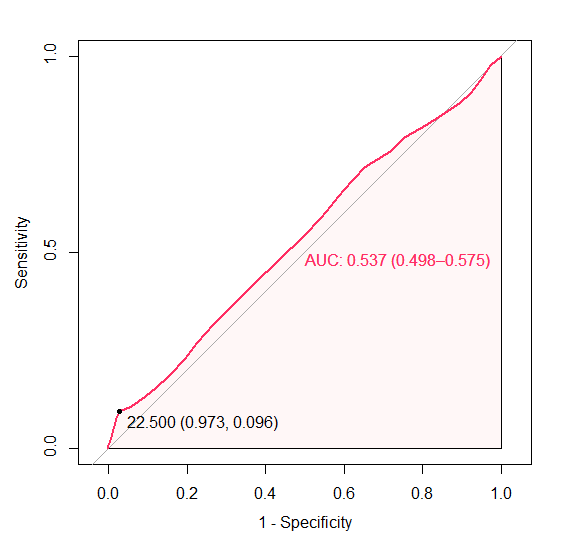


Figure S1. ROC curve analysis of time interval between embryo transfer and infection in predicting clinical pregnancy.The area under the ROC curve was 0.537 (95% CI, 0.498–0.575), while the optimal cutoff value was 22 days (with a sensitivity of 97.3% and specificity of 9.6%)
