# Supplementary material for: SARS-CoV-2 infection is detrimental to pregnancy outcomes after embryo transfer in IVF/ICSI: a prospective cohort study
Source: BMC Med. 2024 Mar 18;22:124. doi: 10.1186/s12916-024-03336-9 (PMC10949839; doi:10.1186/s12916-024-03336-9)
Supplement: Supplementary file 4 — Additional file 4: Table S3. Proportions of the time interval between embryo transfer and infection in the pregnancy group and non-pregnancy group. [file 12916_2024_3336_MOESM4_ESM.docx]

Table S3.Proportions of the time interval between embryo transfer and infection in the **pregnancy group** and non-pregnancy group.

|  | Non-pregnancy group(N = 486) | Pregnancy group(N = 826) | OR | P value |
| --- | --- | --- | --- | --- |
| Time interval between embryo transfer and infection（days） |  |  | 3.76 (1.92-8.24) | <0.001 |
| 0-22d | 97% (320/329) | 90% (511/565) |  |  |
| 23-28d | 3% (9/329) | 10% (54/565) |  |  |
