# Supplementary material for: SARS-CoV-2 infection is detrimental to pregnancy outcomes after embryo transfer in IVF/ICSI: a prospective cohort study
Source: BMC Med. 2024 Mar 18;22:124. doi: 10.1186/s12916-024-03336-9 (PMC10949839; doi:10.1186/s12916-024-03336-9)
Supplement: Supplementary file 5 — Additional file 5: Table S4. SARS-CoV-2 infection symptoms between the pregnancy group and non-pregnancy group. [file 12916_2024_3336_MOESM5_ESM.docx]

Table S4.SARS-CoV-2 infection symptoms between **the pregnancy group** and non-pregnancy group

|  | Non-pregnancy group(N = 486) | Pregnancy group(N = 826) | P value | OR | P value |
| --- | --- | --- | --- | --- | --- |
| Fever | 65% (213/329) | 61% (343/565) | 0.2 | 0.841 ( 0.635 - 1.116 ) | 0.231 |
| Cough/dry cough | 76% (249/329) | 74% (420/565) | 0.7 | 0.931 ( 0.679 - 1.275 ) | 0.654 |
| Sore throat | 37% (121/329) | 38% (215/565) | 0.7 | 1.056 ( 0.797 - 1.399 ) | 0.704 |
| Dizziness/headache | 40% (130/329) | 35% (198/565) | 0.2 | 0.826 ( 0.624 - 1.093 ) | 0.181 |
| Chest tightness/chest pain | 5% (17/329) | 3% (16/565) | 0.074 | 0.535 ( 0.266 - 1.074 ) | 0.078 |
| Muscle soreness/bloating | 47% (153/329) | 45% (253/565) | 0.6 | 0.933 ( 0.71 - 1.225 ) | 0.617 |
| Bone/Arthralgia | 10% (34/329) | 8% (47/565) | 0.3 | 0.787 ( 0.495 - 1.252 ) | 0.312 |
| Diarrhea and abdominal pain | 5% (16/329) | 5% (27/565) | ＞0.9 | 0.982 ( 0.521 - 1.85 ) | 0.955 |
| Nausea and vomiting | 7% (24/329) | 7% (42/565) | ＞0.9 | 1.021 ( 0.606 - 1.718 ) | 0.939 |
| Loss of appetite | 21% (68/329) | 22% (123/565) | 0.7 | 1.068 ( 0.765 - 1.491 ) | 0.698 |
| Fatigue | 21% (68/329) | 18% (102/565) | 0.3 | 0.846 ( 0.6 - 1.191 ) | 0.337 |
| Bitter taste/decreased taste perception | 20% (66/329) | 16% (93/565) | 0.2 | 0.785 ( 0.553 - 1.114 ) | 0.175 |
| Hypoosmia | 9% (29/329) | 8% (44/565) | 0.6 | 0.874 ( 0.535 - 1.426 ) | 0.589 |
| Eye pain/easy tearing/Conjunctivitis | 2% (8/329) | 4% (21/565) | 0.3 | 1.549 ( 0.678 - 3.538 ) | 0.299 |
| Skin symptoms such as Hives and herpes | 1% (4/329) | 1% (7/565) | ＞0.9 | 1.019 ( 0.296 - 3.508 ) | 0.976 |
| Dyspnea | 1% (2/329) | 1% (3/565) | ＞0.9 | 0.873 ( 0.145 - 5.25 ) | 0.882 |
| Severe symptoms such as hypoxemia and acute respiratory distress syndrome | 0% (1/329) | 0% (0/565) | 0.4 | 0 ( 0 - Inf ) | 0.979 |
